# Predicting severe maternal outcomes in a network of sentinel sites in Latin‐American countries

**DOI:** 10.1002/ijgo.14436

**Published:** 2022-09-24

**Authors:** Alicia Aleman, Mercedes Colomar, Valentina Colistro, Gisselle Tomaso, Claudio Sosa, Suzanne Serruya, Luis Andrés de Francisco, Alvaro Ciganda, Bremen De Mucio

**Affiliations:** ^1^ Department of Preventive and Social Medicine School of Medicine Udelar Uruguay; ^2^ Clinical and Research Unit (UNICEM) Montevideo Uruguay; ^3^ Universitat Autonoma de Barcelona Barcelona Spain; ^4^ Latin American Center for Perinatology, Women's Health, and Reproductive Health, (CLAP/WR) Montevideo Uruguay; ^5^ Department of Quantitative Methods School of Medicine Udelar Uruguay; ^6^ Family, Health Promotion, and Life Course, Pan American Health Organization—World Health Organization Washington DC USA

**Keywords:** Latin America, maternal mortality, maternal near miss, potentially life‐threatening conditions, severe maternal outcomes

## Abstract

**Objective:**

This study aimed to determine incidences of potentially life‐threatening conditions (PLTC), maternal near misses (MNM), and maternal deaths (MD) in women who gave birth in participating facilities, and to determine the probability that a pregnancy involving a PLTC would evolve into an MNM and/or an MD.

**Methods:**

This was a multicentric observational study implemented on a maternal network from August 2018 to May 2019 in five Latin‐American countries. We summarized categorical variables as frequencies and continuous variables with median, interquartile range, and standard deviations. Positive and negative likelihood ratios were calculated and multivariate predictive models were built.

**Results:**

There were 33 901 deliveries and miscarriages, of which 8.0% had at least one PLTC and 0.6% had an MNM. Hypertensive disorder was the most frequent condition to evolve into a severe maternal outcome.

**Conclusion:**

Identifying PLTC can help to prevent MNM and MD. The inclusion of these predictors in a real‐time data registration system like the Perinatal Informatic System could work as a surveillance tool for early detection, leading to a reduction in the rate of worsening conditions.

## Synopsis

In Latin‐American hospitals, 8.0% of deliveries/miscarriages involved at least one potentially life‐threatening condition, of which 7.5% evolved into a severe maternal outcome.

## 1 INTRODUCTION

Maternal deaths (MD) are rare events in absolute terms, particularly when they are studied within an individual facility. In this respect, a woman who experiences a severe acute maternal complication and almost dies during pregnancy, childbirth, or the puerperium is considered to have a serious clinical condition called a maternal near miss (MNM).^1^, ^2^, ^3^ MNM share similar characteristics with MD and are a source of information on the barriers that women must overcome to survive. This is a result of health system weaknesses in maternal health care, such as difficulties/delays in accessing healthcare services, inadequate management of obstetrical complications, and failure to provide effective interventions.^4^

Previous research suggested that the study of severe maternal outcomes (SMO), like MNM and MD, as a useful approach to investigating the quality of healthcare systems. This focus can lead to improving women's health care and reducing maternal morbidity and death.^4^ In 2011, the Pan American Health Organization launched a plan to reduce MD, selecting 19 indicators, one of which was MNM surveillance and its evolution at a national level.^5^ However, progress reports in Latin America showed an underreporting of this indicator and a low reliability of data because of this.

Furthermore, from a theoretical standpoint, a woman can only be recognized as an MNM case, retrospectively, as by definition, she needs to have survived a severe complication to become an example of an MNM. However, it was considered clinically useful to have the option of prospectively identifying women presenting a life‐threatening condition. For this reason, WHO proposed to measure some “potentially life‐threatening conditions” (PLTC). These are understood as the first step in a continuous chain of events associated with more severe complications with pregnancy and deserving special attention from healthcare providers, before any organ dysfunction or failure arises (MNM) or the woman dies (MD).^4^ By identifying these conditions and performing well‐known interventions, the chain of events could be prevented from evolving into more severe states.^6^ The implementation of early warning systems could effectively reduce maternal risk of death by capturing the deterioration of women's health before a PLTC occurs.^7^ For instance, an accurate registration of women's obstetrical history would contribute to identifying a high‐risk situation. The frequency with which these PLTC evolve into MNM or MD can be valid indicators to evaluate and improve the quality of health care.^4^

Electronic surveillance systems have been shown to be excellent tools to improve and facilitate complete data reporting and can reduce the time required for data analysis determining the incidence of PLTC, MNM, and MD. Greater benefits are obtained when these surveillance systems are associated with programs that identify risks and propose evidence‐based interventions for the management of severe maternal morbidity.^8^

The main objective of this study was to determine incidences of PLTC, MNM, and MD by the time of discharge in women who gave birth or ended their pregnancy at any gestational age in facilities included in the study. The secondary objective was to determine the probability that a woman with a PLTC would evolve into experiencing an MNM and/or an MD.

## 2 MATERIALS AND METHODS

This was a multicentric observational study implemented on the CLAP maternal network of sentinel sites. The CLAP Network of sentinel sites was composed of seven institutions in five countries from Latin America (Honduras, Guatemala, Dominican Republic, Nicaragua, and Bolivia); four institutions were second level, and three were third‐level maternity hospitals.

All healthcare centers used a data collection system in common called the Perinatal Informatic System (Spanish acronym SIP). SIP is a cost‐free perinatal clinical record system developed by the Pan American Health Organization at CLAP for implementation at healthcare service providers. CLAP developed a specific module for monitoring the burden of SMO (MNM + MD), PLTC, and less severe maternal morbidity during pregnancy, such as birth/miscarriage, and postpartum. The specific module recorded basic demographic data as well as obstetrical history, personal risk factors, current pregnancy information, prenatal controls, birth information, neonatal information, maternal morbidity, interventions, variables to identify cases of MNM, puerperium information, maternal discharge, and contraception.

This specific SIP module (SIP form) has performed well in predicting SMO, so all Latin‐American health facilities that use SIP are expected to improve their management of PLTC and MNM events as well as monitor the quality of health care.^9^ New indicators were proposed as a Mortality index: the number of maternal deaths divided by the number of women with life‐threatening conditions, expressed as a percentage. The higher the index, the more often women with life‐threatening conditions die (proxy rated low quality of care), while the lower the index, the fewer women with life‐threatening conditions die (proxy rated better quality of care).

The PLTC were defined based on De Mucio et al.,^9^ but excluded several laboratory and management variables because of database constraints. Variables from the newly adapted version of the SIP form were included in the morbidity section and are described in Haddad et al.^10^

Women admitted to sentinel sites during pregnancy, delivery, or postpartum that ended a pregnancy (including miscarriages and stillbirths) during the period of this study were included. A SIP form was completed for each woman on site. The number of records registered in SIP was checked against information pulled from hospital records monthly.

As the result of difficulties in following up after discharge, we were not able to assess potential complications among women who gave birth at sentinel sites but might have been admitted to other institutions that did not participate in the CLAP Network.

Data collection started in August 2018 and finished in May 2019. The forms were entered into the SIP hospital database and sent to the data management center monthly. A data management unit performed data quality control and coverage. To recover missing data and solve inconsistencies, quality control reports were sent to the sentinel sites and returned with the requested information.

The research protocol was approved by all participating institutions' institutional review boards and by the Pan American Health Organization Ethics Review Committee (reference number 2018‐04‐0025).

Statisticians determined frequencies of PLTC, MNM, and SMO as well as a description of the evolution into an SMO for each PLTC. Based on this, positive and negative likelihood ratios (LR), and their 95% confidence intervals (CI) were calculated. Multivariate predictive models were built using logistic regression, adjustment for the effect of the cluster design was performed.^11^ R‐statistic software was used for analysis.^12^

This manuscript is reported according to STROBE guidelines for observational studies.^13^

## 3 RESULTS

**FIGURE 1:**
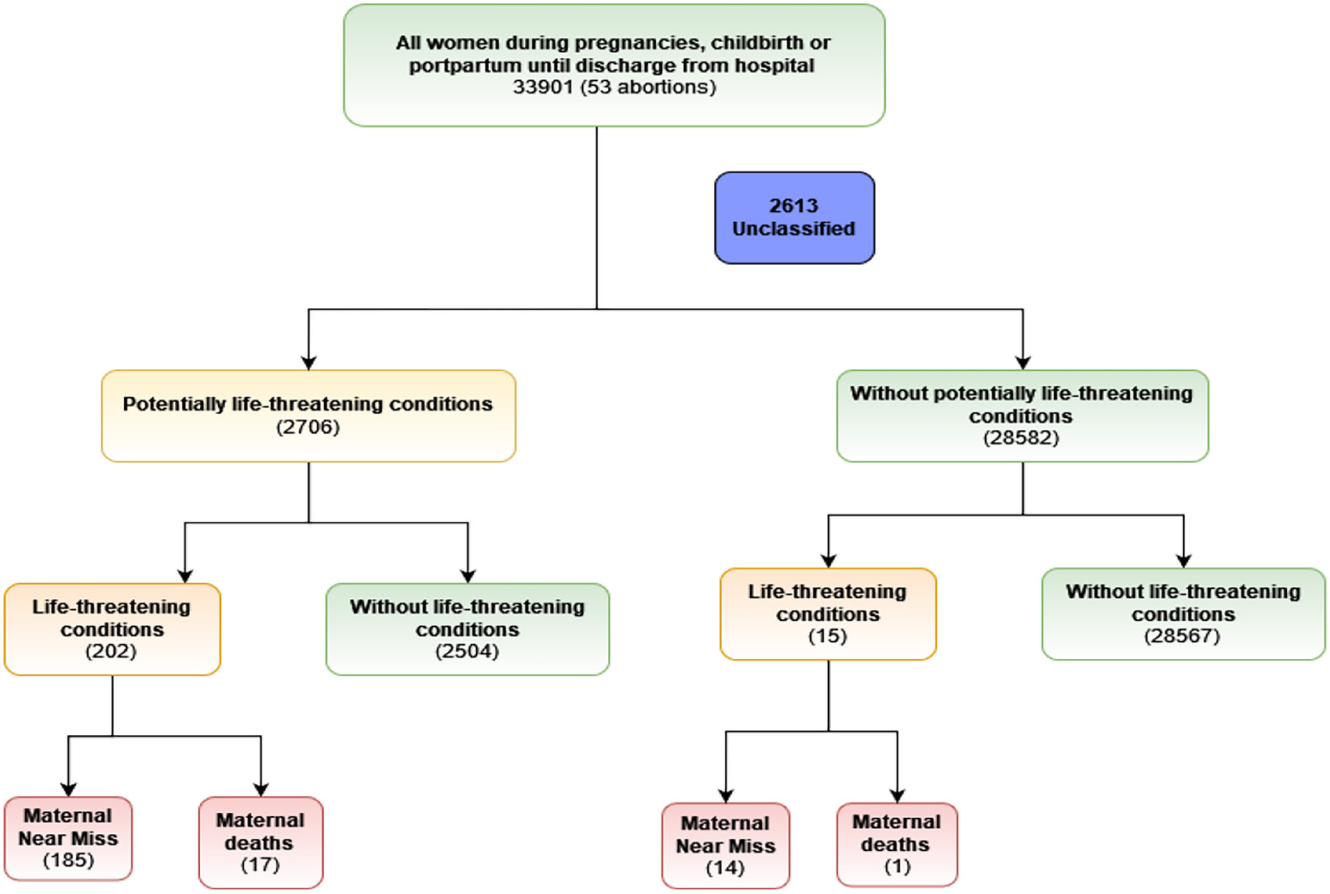
Study flowchart

There were 33 901 deliveries and miscarriages (33 597 live births, 251 stillbirths, and 53 miscarriages) during the period of the study; of which 2706 (8.0%) women had at least one PLTC, 199 (0.6%) had an MNM condition, and 18 (0.0%) resulted in an MD by the time of discharge (Figure 1).

In relation to overall indicators, the maternal mortality ratio was 53.1 per 100 000 records. The incidences of SMO and MNM were 6.40 and 5.87 per 1000 records, respectively. The MNM index per mortality case was 11 to 1 and the mortality index was 9.0%. Most women with an SMO had a previous PLTC. However, there was one woman, who died, and nine women that presented MNM conditions, but did not meet PLTC criteria. The PLTC incidence ratio was: 71.2 per 1000 records for one condition; 7.2 per 1000 for two conditions, and 1.4 per 1000 for three or more conditions.

Distribution of 33 901 deliveries or miscarriages and final outcomes per country are presented in Table 1.

**TABLE 1:** Number of deliveries and miscarriages, and outcomes per country

Country	Deliveries and miscarriages	Number of hospitals	Level of care	Maternal deaths	Maternal near miss	Severe maternal outcome
Bolivia	2437	1	Tertiary	0	16	16
Guatemala	4497	1	Tertiary	8	53	61
Honduras	9201	2	Secondary	0	14	14
Nicaragua	10 102	2	Secondary	1	49	50
Dominican Republic	7664	1	Tertiary	9	67	76

We explored basal characteristics of women with and without SMO. Age, marital status, education, ethnicity, medical background, gestational age, and comorbidities showed statistically significant differences between groups (Table 2). Conditions that were more frequent in the SMO group were maternal age greater than 35 years old, no partner, having only primary or less education, being indigenous or of black ethnicity, having one or more previous cesarean sections (CS), none or fewer than four prenatal visits, induced onset of labor or CS as mode of delivery, and preterm birth. Comorbidities like diabetes, nephropathy, and human immunodeficiency virus infection were also more frequent in the SMO group.

**TABLE 2:** Risk factors for adverse maternal outcomes by women with and without severe maternal outcomes^a^

Characteristics	Deliveries and miscarriages without SMO (*n* = 33 684)	SMO (*n* = 217)	*P* value
Age, year	24.3 ± 6.2	26.6 ± 7.1	<0.01
10–14	385 (1.1)	2 (0.9)	<0.001
15–24	18 748 (56.0)	100 (46.1)	<0.001
25–35	12 401 (37.0)	84 (38.7)	<0.001
> 35	1968 (5.9)	31 (14.3)	<0.001
Marital status			
With partner	31 044 (91.5)	195 (89.8)	<0.001
No partner	1873 (5.6)	14 (7.1)	<0.001
Education			
Primary or less	12 062 (36.1)	96 (46.5)	<0.001
Secondary	18 450 (55.1)	91 (44.6)	<0.001
University	2937 (8.7)	18 (8.8)	0.720
Ethnicity			
White	272 (0.8)	0 (0)	< 0.001
Indigenous	2677 (8)	27 (12.8)	< 0.001
Multiracial (*mestizo*)	28 146 (84.1)	150 (71)	< 0.001
Black	1907 (5.7)	34 (16.1)	< 0.001
Medical background			
No previous CS	27 729 (83.4)	166 (78.7)	<0.001
One previous CS	4165 (12.5)	29 (13.7)	<0.001
Two previous CS	1193 (3.6)	13 (6.2)	<0.001
More than two previous CS	147 (0.5)	3 (1.4)	<0.001
Prenatal care			
None	2671 (8.3)	41 (20.4)	<0.001
One to four visits	12 541 (39.0)	110 (54.7)	<0.001
More tan four visits	16 910 (52.7)	50 (24.9)	<0.001
Mode of delivery			
Spontaneous	24 933 (74.4)	131 (60.9)	<0.001
Induced	1575 (4.7)	15 (7.0)	<0.001
Elective CS	7001 (20.9)	69 (32.1)	<0.001
Gestational age, week			
< 32	548 (1.6)	38 (17.5)	<0.001
32–36	2400 (7.2)	51 (23.5)	<0.001
> 36	3451 (91.2)	128 (59)	<0.001
Comorbidities			
Diabetes	169 (0.5)	2 (0.92)	<0.001
Hypertension	442 (1.3)	8 (0.04)	<0.001
Heart disease	44 (0.1)	0 (0)	<0.001
Nephropathy	13 (0.03)	1 (0.46)	<0.001
HIV	100 (0.3)	1 (0.46)	<0.001
Others	343 (1.00)	8 (3.70)	<0.001

Abbreviations: CS, cesarean section; HIV, human immunodeficiency virus; SMO, severe maternal outcomes.

^a^ Data are presented as mean ± standard deviation or as number (percentage).

In Table S1, PLTC frequencies and their predictive capacity to evolve into SMO are presented. For categories of PLTC (developed during pregnancy), hypertensive disorders were the most frequent type of condition (1586 women, 4.6%) followed by hemorrhage (779, 2.3%), infections (572, 7.7%), neurologic disorders (69, 0.2%), and others (25, 0.1%) (not mathematically strong conditions to be disaggregated). Severe pre‐eclampsia was present in 1508 women in the database (4.4%) and 82 of these evolved into an SMO (5.4%). HELLP (hemolysis, elevated liver enzymes and low platelet count) syndrome was the second most frequent PLTC among hypertensive disorders with 265 women in the database having this condition (0.78%), of whom 66 (25%) evolved into an SMO.

PLTC with fair diagnostic capacity to predict SMO (LR+ ≥10 to 19) were placental abruption and placenta previa. Eclampsia, HELLP syndrome, ectopic pregnancy, lacerations, retained products of conception, uterine atony, endometritis, seizures, systemic inflammatory response syndrome, and sepsis had good diagnostic capacities to predict SMO (LR+ ≥20 to 79). Altered state of consciousness, placenta accreta, postpartum hemorrhage, uterine rupture, pneumonia, and disseminated intravascular coagulopathy all had very good diagnostic capacities to predict SMO when positive (LR+ ≥80). Negative likelihood ratios for all PLTC were higher than 0.10, which means that the absence of a PLTC is not a good predictor of the absence of SMO (see Table S1).

In the multivariate analysis that included all PLTC, only 17 conditions remained in the model (*P* ≤ 0.05) (see Table 3). Women with coagulation defects had a 266‐fold likelihood of developing an SMO: adjusted odds ratio (OR) 266 (95% CI 34–2086). This was followed by altered state of consciousness (OR 102, 95% CI 11–962), pulmonary thromboembolism (OR 97, 95% CI 3–1326), pneumonia (OR 54, 95% CI 8–348), and uterine rupture (OR 42, 95% CI 6–305). Adjusted OR for remaining PLTC to be significantly associated with SMO ranged from 2.44 (other infections) to 29.68 (placenta accreta). Testing for the effect of the cluster design was performed but no correlation among clusters was found.

**TABLE 3:** Multivariate adusted logistic regression models all potentially life‐threatening conditions^a^

Disorder	Potentially life‐threatening conditions	Severe maternal outcome			
		Estimate	*Z*	*P* value	OR (95% CI)
Coagulation disorders	Pulmonary thromboembolism	4.08 ± 1.59	2.57	0.010	59.07 (2.63–1326.27)
Coagulation disorders	Coagulation defects	5.58 ± 1.05	5.31	0,000	265.87 (33.89–2085.93)
Hemorrhagic disorders	Placenta accreta	3.39 ± 1.19	2.84	0.004	29.68 (2.87–307.07)
Hemorrhagic disorders	Postpartum hemorrhage	2.87 ± 0.31	9.12	0.000	17.56 (9.49–32.51)
Hemorrhagic disorders	Placental abruption	1.85 ± 0.49	3.78	0.001	6.36 (2.43–16.59)
Hemorrhagic disorders	Uterine rupture	3.74 ± 1.01	3.71	0.000	42.16 (5.83–304.8)
Hemorrhagic disorders	Ectopic pregnancy	3.1 ± 1.49	2.08	0.038	22.19 (1.19–413.56)
Hemorrhagic disorders	Uterine atony	2.22 ± 0.32	6.89	0.000	9.19 (4.89–17.29)
Hemorrhagic disorders	Lacerations	1.78 ± 0.66	2.68	0.007	5.91 (1.61–21.64)
Hemorrhagic disorders	Ovular remains	1.84 ± 0.6	3.06	0.002	6.32 (1.94–20.59)
Hypertensive disorders	HELLP syndrome	2.48 ± 0.32	7.68	0.000	11.94 (6.34–22.49)
Hypertensive disorders	Eclampsia	2.27 ± 0.55	4.14	0.000	9.65 (3.3–28.21)
Hypertensive disorders	Severe pre‐eclampsia	1.58 ± 0.27	5.76	0,000	4.84 (2.83–8.28)
Infections	Other infections	0.89 ± 0.44	2.02	0.043	2.44 (1.03–5.79)
Infections	Endometritis	2.4 ± 0.79	3.03	0.002	10.98 (2.33–51.73)
Infections	Pneumonia	4 ± 0.95	4.22	0.000	54.37 (8.49–348.35)
Neurologic disorders	Altered state of consciousness	4.62 ± 1.15	4.03	0.001	101.69 (10.75–961.77)

Abbreviations: CI, confidence interval; HELLP, hemolysis, elevated liver enzymes and low platelet count; OR, odds ratio.

^a^ Data are presented as mean ± standard error unless otherwise stated.

## 4 DISCUSSION

This study was able to determine the incidence of PLTC, MNM, and MD up to the time of hospital discharge for women receiving delivery care at a network of maternity hospitals, in five Latin‐American countries. Additionally, results confirmed that a number of pregnancy‐related PLTC were found to be highly associated with MNM and/or MD.

The MD ratio in our study was 53.1 per 100 000 births. Two countries included in this study are classified according to the World Bank as upper‐middle income (Dominican Republic and Guatemala) and three as lower‐middle income (Bolivia, Honduras, and Nicaragua).^14^, ^15^ A systematic review that included 62 papers, reported a median MD ratio of 306 per 100 000 live births (interquartile range [IQR] 162–666) in lower‐middle‐income countries; 163 per 100 000 live births (IQR 52–367) in middle‐income countries; and 62 per 100 000 live births (IQR 9–105) in higher‐middle‐income countries.^16^ Even though our figures are included in the interquartile interval of ratios reported by Heitkamp et al.,^16^ there are differences in our study's population that may explain variations with the point estimate. In this study, we included deaths until time of discharge related to delivery or miscarriage. We might be missing deaths occurring from discharge to day 42. Another possible explanation for this difference is that some of the maternity hospitals included did not contribute to the overall figure of maternal deaths, possibly because of small numbers of deliveries or references to tertiary level hospitals.

Incidence of MNM in our study was 5.9 per 1000 births. This ratio was lower than that reported by De Mucio et al.^9^ in a population of Latin‐American countries (12.3 per 1000 births), by Heitkamp et al.^16^ in middle‐income countries of several continents (9.6 per 1000 births), as well as by the Network for Surveillance of Severe Maternal Morbidity of Brazil (9.3 per 1000 births).^17^ There are several potential explanations for these differences.

Primarily, there is no standardized MNM definition. The WHO definition is the most commonly accepted, “a woman who nearly died but survived a complication that occurred during pregnancy, birth, or within 42 days of termination of pregnancy”.^4^ In practical terms, an MNM is identified when a woman develops signs of organ dysfunction as per a number of clinical, laboratory, or management criteria.^4^ However, there is a limitation in the applicability of these criteria in settings from low‐ or middle‐income countries, where routine data collection and registration do not include certain laboratory results or management procedures. Second, differences in the estimation of MNM could be the number of events included in our study. We included 10‐fold more deliveries and miscarriages than did De Mucio et al.,^9^ which could supply a more accurate estimation of this indicator. Finally, our study included women from second‐level hospitals (four out of seven centers) which might not accept women at risk of requiring intensive care. These women may have been referred to a more complex level of care, which could have caused an under‐recording of MNM cases. Both Cecatti et al.^17^ and De Mucio et al.,^9^ included only women from third‐level reference hospitals.

The incidence of PLTC was 71 per 1000 births. Comparability with other countries was difficult because of the heterogeneity in the definition of PLTC. In the Brazilian network, PLTC was 99.8 per 1000 births according to WHO criteria.^17^ We detected more than one PLTC in 8.6 per 1000 deliveries and miscarriages, whereas the Brazilian network had 5.1 per 1000.

The PLTC group most frequently reported in our study was hypertensive disorders, followed by hemorrhages and infections, which was similar to findings reported by Cecatti et al.^17^ These groups of diseases are related to the development of MNM in our study as in other middle‐income countries.^17^, ^18^, ^19^ In our univariate analysis, PLTC included in those three groups were particularly good predictors of SMO, with likely positive ratios. The most predictive PLTC were altered state of consciousness (LR+, 465), placenta accreta (LR+, 346), coagulation disorders (LR+, 309), and uterine rupture (LR+, 115), as was seen in a previous study.^9^

After designing a multiple regression model, the highest association to SMO was with coagulation disorders (OR 266, 95% CI 34–2086) and with altered state of consciousness (OR 102, 95% CI 11–962). The predictive ability of the PLTC uterine rupture or placenta accreta decreased, but not to a level that stopped them from being reliable predictors (Table 3). Other good predictor conditions (OR ≥ 10) were: suspicion of ectopic pregnancy (OR 22), postpartum hemorrhage (OR 18), HELLP syndrome (OR 12), endometritis (OR 11), and eclampsia (OR 10). Although confidence intervals were broad, the magnitude of the point estimates was too high to ignore, and the association was statistically significant in all cases.

An SMO can be conceptualized as a continuum of complications with increasing severity starting in a PLTC and ending in an MD. The objective of this study was to identify the earliest and most predictive conditions to avoid SMO and to “turn on” alerts for action. The delay in accessing appropriate care after detecting a predictive condition may be one of the main problems to solve. A structured triage process to appropriately refer women and decrease the time it takes to see senior‐level providers were two successful strategies already implemented.^20^, ^21^ Indeed, monitoring the quality of care within institutions as routine assessments of MNM cases could also help. Moreover, the analysis of critically ill women that survived a PLTC is a useful tool to identify breakdown areas in the healthcare system.^4^

The present study has a number of limitations. The data collection period was less than a year, so recruitment of women in some locations was limited. The inclusion of second‐level hospitals may have played a role in the underestimation of PLTC, MNM, and MD as it is likely that women with severe conditions were referred to third level hospitals and were therefore not included in our database. Finally, the women included in this study were tracked until discharge but NM and MD definitions include events that occur up to 42 days after delivery. Our figures are not a perfect estimation of SMO, but could be considered good proxy indicators of maternal morbidity and mortality in five low‐middle‐income countries in the Latin‐American region. This study contributes data collection and determination of potential predictive factors for SMO.

The present study also has several strengths. First, we included countries where this health problem has not been well studied. Second, our study sample size is larger than that of earlier studies implemented in the region. Third, it uses a pragmatic approach, as the data collection was carried out with a registration system broadly implemented across the region, allowing comparability among Latin‐American countries.

In conclusion, identifying predictors for adverse maternal outcomes can make avoiding cases of NM and preventing MD easier. The inclusion of these predictors in a real‐time data registration system, like SIP Plus, could work as a surveillance tool for the early detection and prevention of challenging situations during pregnancy and delivery. Then these conditions, when present, would immediately trigger intervention protocols. Ideally, the detection of less severe conditions could also trigger rapid responses to avoid progression to SMO. In order to do this, there are two aspects that need to be strengthened: training healthcare staff to recognize red flags and having the necessary measures in place for an appropriate, rapid response.

Active surveillance of SMO has been neglected regionally in this health field. There is a need for a call to action to raise awareness, make it visible, and face this problem.

## AUTHOR CONTRIBUTIONS

BDM and CS conceived the study; AA, GT, BDM, VC, and MC, wrote the original draft and searched the literature; AC validated the data and performed quality control; VC and MC performed data analysis; AA, GT, BDM, and CS designed the study, interpreted the data, and contributed to the writing; BDM, CS, LAdF, and SS contributed to the writing, reviewed the manuscript, and made substantial contributions.

## CONFLICT OF INTEREST

The authors declare that they have no conflicts of interest. The authors, as staff members of the Pan American and World Health Organization, are responsible for the views expressed in this publication, which do not necessarily represent the decisions or policies of the Pan American Health Organization.

## Supporting information


Table S1

## Data Availability

Research data are not shared.

## References

[1] Zeeman GG , Wendel GDJ , Cunningham FG . A blueprint for obstetric critical care. Am J Obstet Gynecol. 2003;188:532‐536. doi:10.1067/mob.2003.95 12592267

[2] Say L , Pattinson RC , Gülmezoglu AM . WHO systematic review of maternal morbidity and mortality: the prevalence of severe acute maternal morbidity (near miss). Reprod Health. 2004;1:3. doi:10.1186/1742-4755-1-3 15357863 PMC516581

[3] Pattinson RC , Hall M . Near misses: a useful adjunct to maternal death enquiries. Br Med Bull. 2003;67:231‐243. doi:10.1093/bmb/ldg007 14711767

[4] Say L , Souza JP , Pattinson RC . Maternal near miss‐‐towards a standard tool for monitoring quality of maternal health care. Best Pract Res Clin Obstet Gynaecol. 2009;23:287‐296. doi:10.1016/j.bpobgyn.2009.01.007 19303368

[5] Organización Panamericana de la Salud . Plan de acción para acelerar la reducción de la mortalidad materna y la morbilidad materna grave: Informe final. 162.a sesión del Comité Ejecutivo; del 18 al 22 de junio del 2018. (documento CE162/INF/12) Washington, DC: OPS; 2018 [consultado el 16 de may]. Washington, DC: 2018.

[6] Campbell OMR , Graham WJ . Strategies for reducing maternal mortality: getting on with what works. Lancet (London, England). 2006;368:1284‐1299. doi:10.1016/S0140-6736(06)69381-1 17027735

[7] Friedman AM , Campbell ML , Kline CR , Wiesner S , D'Alton ME , Shields LE . Implementing obstetric early warning systems. AJP Rep. 2018;8:e79‐e84. doi:10.1055/s-0038-1641569 29686937 PMC5910060

[8] World Health Organization (WHO) . Protocol for the evaluation of epidemiological surveillance systems 1997.

[9] De Mucio B , Abalos E , Cuesta C , et al. Maternal near miss and predictive ability of potentially life‐threatening conditions at selected maternity hospitals in Latin America. Reprod Health. 2016;13:134. doi:10.1186/s12978-016-0250-9 27814759 PMC5097347

[10] Haddad SM , Cecatti JG , Parpinelli MA , et al. From planning to practice: building the national network for the surveillance of severe maternal morbidity. BMC Public Health. 2011;11:283. doi:10.1186/1471-2458-11-283 21549009 PMC3101659

[11] Haddad SM , Sousa MH , Cecatti JG , Parpinelli MA , Costa ML , Souza JP . Intraclass correlation coefficients in the Brazilian network for surveillance of severe maternal morbidity study. BMC Pregnancy Childbirth. 2012;12:101. doi:10.1186/1471-2393-12-101 22998520 PMC3570276

[12] R Core Team . R: A language and environment for statistical computing. R. Foundation for Statistical Computing, Vienna, Austria. 2019. https://www.R‐project.org/ (accessed March 15, 2022).

[13] Vandenbroucke JP , von Elm E , Altman DG , et al. Strengthening the reporting of observational studies in epidemiology (STROBE): explanation and elaboration. Int J Surg. 2014;12:1500‐1524. doi:10.1016/j.ijsu.2014.07.014 25046751

[14] World Bank . Databank: upper middle income 2020. https://data.worldbank.org/income‐level/upper‐middle‐income (accessed December 21, 2021).

[15] World bank data team . Databank: lower middle income 2020. https://data.worldbank.org/income‐level/lower‐middle‐income (accessed December 21, 2021).

[16] Heitkamp A , Meulenbroek A , van Roosmalen J , et al. Maternal mortality: near‐miss events in middle‐income countries, a systematic review. Bull World Health Organ. 2021;99:693‐707F. doi:10.2471/BLT.21.285945 34621087 PMC8477432

[17] Cecatti JG , Costa ML , Haddad SM , et al. Network for surveillance of severe maternal morbidity: a powerful national collaboration generating data on maternal health outcomes and care. BJOG. 2016;123:946‐953. doi:10.1111/1471-0528.13614 26412586

[18] Lima HMP , Carvalho FHC , Feitosa FEL , Nunes GC . Factors associated with maternal mortality among patients meeting criteria of severe maternal morbidity and near miss. Int J Gynaecol Obstet off Organ Int Fed Gynaecol Obstet. 2017;136:337‐343. doi:10.1002/ijgo.12077 28099693

[19] Madeiro AP , Rufino AC , Lacerda ÉZG , Brasil LG . Incidence and determinants of severe maternal morbidity: a transversal study in a referral hospital in Teresina, Piaui, Brazil. BMC Pregnancy Childbirth. 2015;15:210. doi:10.1186/s12884-015-0648-3 26347370 PMC4562200

[20] Edson W , Burkhalter B , Harvey S , Boucar M , Djibrina S , Hermida J , et al. Safe motherhood studies—Timeliness of in‐hospital care for treating obstetric emergencies: Results from Benin, Ecuador, Jamaica, and Rwanda. Operations Research Results. Published for the U.S. Agency for International Development (USAID) by QAP. 2006.

[21] Calvello EJ , Skog AP , Tenner AG , Wallis LA . Applying the lessons of maternal mortality reduction to global emergency health. Bull World Health Organ. 2015;93:417‐423. doi:10.2471/BLT.14.146571 26240463 PMC4450708

